# Age-Related Changes in BOLD Activation Pattern in Phonemic Fluency Paradigm: An Investigation of Activation, Functional Connectivity and Psychophysiological Interactions

**DOI:** 10.3389/fnagi.2016.00110

**Published:** 2016-05-23

**Authors:** Christian La, Camille Garcia-Ramos, Veena A. Nair, Timothy B. Meier, Dorothy Farrar-Edwards, Rasmus Birn, Mary E. Meyerand, Vivek Prabhakaran

**Affiliations:** ^1^Neuroscience Training Program, University of Wisconsin-MadisonMadison, WI, USA; ^2^Department of Radiology, University of Wisconsin-MadisonMadison, WI, USA; ^3^Department of Medical Physics, University of Wisconsin-MadisonMadison, WI, USA; ^4^Department of Neurosurgery, Medical College of WisconsinMilwaukee, WI, USA; ^5^Department of Kinesiology, University of Wisconsin-MadisonMadison, WI, USA; ^6^Department of Psychiatry, University of Wisconsin-MadisonMadison, WI, USA; ^7^Department of Biomedical Engineering, University of Wisconsin-MadisonMadison, WI, USA

**Keywords:** BOLD fMRI, functional connectivity, normal aging, psychophysiological interactions, task positive network, DMN

## Abstract

Healthy aging is associated with decline of cognitive functions. However, even before those declines become noticeable, the neural architecture underlying those mechanisms has undergone considerable restructuring and reorganization. During performance of a cognitive task, not only have the task-relevant networks demonstrated reorganization with aging, which occurs primarily by recruitment of additional areas to preserve performance, but the task-irrelevant network of the “default-mode” network (DMN), which is normally deactivated during task performance, has also consistently shown reduction of this deactivation with aging. Here, we revisited those age-related changes in task-relevant (i.e., language system) and task-irrelevant (i.e., DMN) systems with a language production paradigm in terms of task-induced activation/deactivation, functional connectivity, and context-dependent correlations between the two systems. Our task fMRI data demonstrated a late increase in cortical recruitment in terms of extent of activation, only observable in our older healthy adult group, when compared to the younger healthy adult group, with recruitment of the contralateral hemisphere, but also other regions from the network previously underutilized. Our middle-aged individuals, when compared to the younger healthy adult group, presented lower levels of activation intensity and connectivity strength, with no recruitment of additional regions, possibly reflecting an initial, uncompensated, network decline. In contrast, the DMN presented a gradual decrease in deactivation intensity and deactivation extent (i.e., low in the middle-aged, and lower in the old) and similar gradual reduction of functional connectivity within the network, with no compensation. The patterns of age-related changes in the task-relevant system and DMN are incongruent with the previously suggested notion of anti-correlation of the two systems. The context-dependent correlation by psycho-physiological interaction (PPI) analysis demonstrated an independence of these two systems, with the onset of task not influencing the correlation between the two systems. Our results suggest that the language network and the DMN may be non-dependent systems, potentially correlated through the re-allocation of cortical resources, and that aging may affect those two systems differently.

## Introduction

The median age of the population in the US and other developed nations is rapidly increasing, reinforcing the need for a better understanding of age-related brain processes and mechanisms. Even before the appearance of noticeable decline in cognitive abilities, commonly associated with advanced age, the neural architectures underlying these processes and functions may have already undergone considerable changes (Paulsen et al., 2004; Hampel et al., 2008; Callaghan et al., 2014). Cognitive neuroimaging studies have consistently shown age-related cortical network re-structuring, more specifically a reduction of hemispheric specialization for more bilateral activation (Reuter-Lorenz et al., 2000; Cabeza, 2002). This phenomenon known as the hemispheric asymmetry reduction in older adults (HAROLD) model (Cabeza, 2002) is well documented with studies using various imaging modalities, including functional MRI (Cabeza, 2002), electro-encephalography (Bellis et al., 2000), and near infrared spectroscopy (Herrmann et al., 2006).

Various groups have also put forward cognitive constructs of aging such as the “compensation related utilization of neural circuits hypothesis” (CRUNCH) (Reuter-Lorenz and Lustig, 2005; Reuter-Lorenz and Cappell, 2008) and the “scaffolding theory of aging and cognition” (STAC) (Park and Reuter-Lorenz, 2009). These constructs posit the recruitment of proximal and/or distal brain structures for compensation in the older adults in the event of a decline of the functional neuronal unit. This ultimately forges an alternative neural circuit to preserve performance. These hypotheses are further supported by the reduction of functional specialization, or “dedifferentiation” in aging (Baltes and Lindenberger, 1997; Li et al., 2001; Park et al., 2004), where the functional unit exhibits reduced functional specificity in order to participate in the recruitment in the goal-directed behavior. The phenomenon has been documented in various systems: ventral visual system (Park et al., 2004, 2012), motor system (Carp et al., 2011a; Bernard and Seidler, 2012) as well as perceptual and cognitive systems (Carp et al., 2011b).

In terms of the language system, young adults demonstrate a left hemisphere lateralized activation pattern. A number of studies have shown a reduction of lateralization in the older compared with younger adults. During an overt picture-naming task, it was demonstrated that larger frontal network activation as well as a reduction of lateralization in the elderly compared with younger adults was found, as evidenced by increased right frontal activation (homolog of Broca's area, BA 45; anterior inferior frontal gyrus, IFG; anterior cingulate) (Wierenga et al., 2008). Moreover, Meinzer et al. (2009) demonstrated recruitment of the contralateral homolog in two overt verbal fluency tasks (semantic and phonemic). While performance during the phonemic task was comparable for both age groups, the performance during the semantic task in the old group was negatively correlated with activation of the right frontal activity (i.e., the right contralateral homolog).

In addition to task-relevant network, changes have been observed in the “default-mode” network (DMN). Comprised of the precuneus/posterior cingulate cortex (pC/PCC) complex, the medial prefrontal cortex (mPFC), and bilateral inferior parietal lobules (IPL) at its core, the DMN is a set of regions previously observed to consistently exhibit deactivation during active task and activation during the “resting” condition (Shulman et al., 1997; Binder et al., 1999; Gusnard et al., 2001; Raichle et al., 2001; Fox and Raichle, 2007). For the purpose of this study, *rest* is defined as a condition where no active participation in a task is required. This network has been suggested to hold a role in mind-wandering (Raichle et al., 2001; Ochsner et al., 2004; Schmitz et al., 2004; Mason et al., 2007; Buckner et al., 2008; Qin and Northoff, 2011), directing resources away from goal-directed processes, and thus may be anti-correlated to task-positive networks (TPNs; Fox et al., 2009). However, others have argued that the DMN is involved in goal-directed behavior itself with activation in response to introspection and self-referential processes (Ochsner et al., 2004; Schmitz et al., 2004; Buckner et al., 2008; Qin and Northoff, 2011), hence reducing the significance of the previously suggested anti-correlation.

With aging, altered patterns of deactivation in the DMN during task have been documented and have been proposed to be related to declining resources, difficulties with resource allocation, or both (Persson et al., 2007). Generally suppressed during the performance of many tasks including language tasks, modifications of this default-mode network with aging have led to the observation of a failure in the disengagement from default-mode processes, resulting in a reduction to demand sensitivity, and thus interfering with proper task performance (Persson et al., 2007). Furthermore, activity during different phases of a working memory paradigm (encoding and recognition) exhibited similar reduced deactivation, suggesting an age-related reduction in the ability to suspend non-task-related processes in the older group (Persson et al., 2007). Moreover, Meinzer et al. (2012) documented an apparent reduction in DMN deactivation during both semantic and phonemic verbal fluency task in the older adults in comparison to the younger participants, in association with the additional recruitment of the contralateral right frontal homolog, suggesting a possible interplay between TPN and DMN systems.

Currently, most neuroimaging studies using task fMRI have focused on the investigation of patterns of the task-induced blood-oxygen level-dependent (BOLD) activation. However, it has been further hypothesized that aside from regional activation changes, alterations in functional brain connectivity also occurs, driving the observed age-related deficits. Functional brain connectivity is a measure often used in the analysis of resting-state fMRI, but is applicable in task-fMRI as well, measuring the synchronicity of the task-induced BOLD activity fluctuations between regions. Some evidence of decrease in task-related connectivity in aging can be found in the working memory literature, and was associated with reduction of cognitive performance (Daselaar et al., 2006; Nagel et al., 2011). Differences in brain connectivity have been observed when levels of brain activity between the two populations (young vs. old) were the same (Madden et al., 2010), suggesting a reorganization of the functional framework to precede the change in pattern of activation. Brain connectivity changes related with aging are thought to be useful in order to interpret functional reorganization in the context of the models mentioned above of functional brain compensation and dedifferentiation (Sala-Llonch et al., 2015).

Additionally, a psycho-physiological interactions (PPI) analysis (Friston et al., 1997) can be implemented to assess the context-dependent correlations; the influence of condition (i.e., the onset of the task) on correlations of the oscillations between distal regions or systems. This method would allow for an assessment of interaction between the systems (i.e., the language and DMN systems), and whether age could change such interactions. With the implementation of a modified verbal fluency task, we reviewed the age-related functional changes in regions within the network, but also their interaction with regions from other networks, such as regions of the DMN. Specifically, we investigated: (1) the intensity and extent of cortical activation, (2) the intensity and extent of task functional connectivity; but also (3) a characterization of the interaction, or lack thereof, between the language network and the DMN during task performance in different age populations with a context-dependent correlation analysis. Additionally, we provided an assessment of activity within two non-language systems with minimum implication in a language production task, which were the auditory and motor networks. In this study, we hypothesize that reorganization of the language-relevant system would be implemented through a recruitment of predisposed brain regions within the functional network, a recruitment which can be characterized by intensity/extent of activation and strength/extent of connectivity. Such measures would provide complementary information in the characterization of age-related differences in the cortical system. Furthermore, we hypothesize that these two systems may undergo changes as part of two independent, but co-occurring events.

## Materials and methods

### Subjects

Fifty-seven individuals were recruited to participate in this study: 20 young adult subjects (≤ 30 years old, mean = 23.0 ± 3.4 years), 18 middle-aged subjects (50–59 years old, mean = 54.0 ± 2.9 years) and 19 older adult subjects (≥60 years old, mean = 63.7 ± 5.2 years). The group of young adults served as a control group for the two other groups (middle-aged and old) in this assessment of aging. The middle-aged subjects between the age of 50 and 59 served as an intermediate group, between the young and older adult groups. All participants were healthy, right-handed [according to the Edinburgh handedness inventory (Oldfield, 1971)], and with no history of neurological or psychiatric disorders. Further details about demographic information for subjects can be found in Table 1. Each individual received a brief out-of-scanner neuropsychological assessment battery, as well as a fMRI session, comprising of a high-resolution structural MRI scan and a task fMRI scan utilizing a verbal fluency, language production, paradigm. All participants were recruited and consented for study participation in full compliance to a protocol approved by the University of Wisconsin-Madison Health Sciences Institutional Review Board (IRB).

**Table 1:** **Subjects' demographic and behavioral characteristics**.

**Characteristics of participants**	**Age ≤ 30**	**Age 50–59**	**Age ≥ 60**
Age (years)	23.0 ± 3.4	54.0 ± 2.9	63.7 ± 5.2
Gender (male/female)	12/8	9/9	11/8
Education (years)	16.6 ± 2.4	18.0 ± 3.4	17.2 ± 2.8
Scanning protocol (A/B)^†^	10/10	9/9	11/8
Verbal fluency score-raw	41.6 ± 13.8	48.3 ± 13.6	48.4 ± 15.0
Verbal fluency score-normalized	−0.025 ± 1.08	0.027 ± 1.05	0 ± 1

† *Two scanners with slight deviation in scanning parameters were used. Details can be found in the method section*.

### MRI acquisition

All neuroimaging data was acquired at the University of Wisconsin-Madison with two identical 3.0-Tesla GE Discovery MR750 scanners (GE Healthcare, Waukesha, WI) equipped with an 8-channel phased array head coil. Task fMRI scans were obtained using single-shot echo-planar T2^*^-weighted with 40 slices. Parameters of scan differed slightly across the two scanners with one having TR = 2 s, TE = 30 ms, flip angle = 75°, FOV = 240 mm, 3.75 × 3.75 × 4 mm voxel (scanning protocol A), and the other TR = 2.6 s, TE = 22 ms, FOV = 224 mm, flip angle = 60°, isotropic 3.5 mm voxel resolution, both with matrix size of 64 × 64 (scanning protocol B). Scanner differences were accounted for in the analysis by integration of scanner covariate as part of the regression model. The proportion of participants in each scanning protocol is detailed in Table 1. High-resolution 3D T1-weighted BRAVO, IR-prepared FSPGR (Fast Spoiled Gradient Recalled Echo), MRI sequence with 156 contiguous axial slices was performed for each participant using the following sequence parameters: TR = 8.13 ms, TE = 3.18 ms, TI = 450 ms; FOV = 256 mm; matrix size = 256 × 256; slice thickness = 1.0 mm; flip angle = 12°. Subjects were provided earplugs and foam paddings to attenuate scanner noise and minimize head motion. Subjects were instructed to hold still and minimize head motion before the start of each scan.

### Experimental task

**Figure 1:**
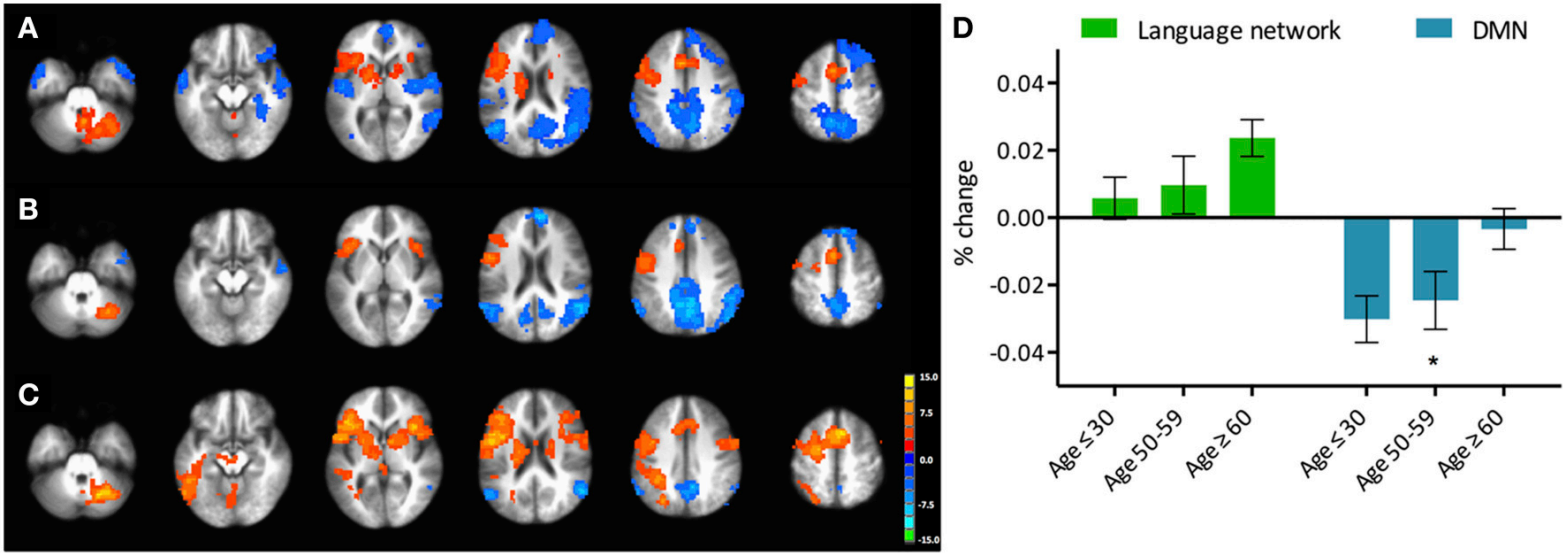
**Phonemic verbal fluency task activation maps and quantitative change in activity**. (Left) Activation maps in **(A)** young (≤ 30 years), **(B)** middle-aged (50–59 years), and **(C)** older adults (≥60 years) subjects. Weakening of task-positive and task-negative activation in the older subjects is evident. Cluster-wise corrected at *p* < 0.005, α = 0.05, minimum cluster size = 105 contiguous voxels. Maps are provided following neurological convention, left is on left. (Right, **D**) Task-positive (green) percent-change showing slow increase with age, and task-negative (blue) showing abrupt decline in the group of participants over 60 years. Statistical values are generated using a one-way ANOVA test (^*^Significant at *p* < 0.05).

A phonemic verbal fluency task (COWAT Benton and Hamsher, 1976) was chosen for this study as it has been shown to produce robust left hemisphere lateralized activation of regions in young healthy adults (Costafreda et al., 2006; Prabhakaran et al., 2007; Meinzer et al., 2009). To minimize motion artifacts, we opted for a modified version of the task, where participants were instructed to think, but not to vocalize words that start with a letter shown in the center of their visual field. Though subjects' compliance to task demand cannot be verified because of the non-vocalization, compliance was verified by verbal assessment at the end of the scan session. Additionally, data collected over those sessions produced mapping of the language production network (Figure 1). Out-of-scanner COWAT was also implemented as part of the behavioral assessment, providing training for the in-scanner task. The in-scanner verbal fluency (VF) task paradigm was implemented using a block design of 20 s task blocks interleaved and padded with 20 s of rest at each end of the run. The duration of the phonemic verbal fluency fMRI scan totaled for a length of 3 min, after which the subject were verbally asked whether he/she was able to properly perform the task. Subjects were reminded to perform as proficient as they can in word generation when the letter appears on the screen.

### Data preprocessing

AFNI software package (http://afni.nimh.nih.gov; Cox, 1996) was used for reconstruction, preprocessing and analysis of the MR images. Preprocessing steps for task fMRI included the removal of the first three volumes, motion correction, slice-timing correction, 6-mm full width at half maximum (FWHM) Gaussian smoothing, and percent signal change normalization of the BOLD signal. Data from subjects exceeding 2-mm motion in any direction were discarded from analysis. Functional and anatomical images were then aligned and transformed to Talairach coordinate system (Talairach and Tournoux, 1988). The subsequent preprocessed functional images underwent regression analysis with the verbal fluency task onset time-file serving as a regression model. The six parameters of rigid motion also were used as regressors of no-interest. To account for the difference in brain volume of the participants, and to address the concern of more interpolation in the older populations during normalization to template space, measures of intra-cortical brain volume, extracted using FSL's *fast* program [Functional Magnetic Resonance Imaging of the Brain (FMRIB) Software Library (FSL)], were used as covariates in the analysis.

### Task-induced BOLD activation analysis

Analysis of task-induced activation included within-group and between-group comparisons of normalized whole-brain BOLD activation maps, with scanner site and intra-cortical volumes acting as covariates. These analyses were thresholded at cluster-wise corrected individual *p*-value of 0.005 with alpha significance level of 0.05, minimum cluster-size of 105 contiguous voxels, as determined by Monte Carlo simulation with the use of AFNI's *3dClustSim*. Region-of-interest (ROI) analysis was also performed by extracting mean normalized intensity values over each set of anatomical masks (i.e., the language mask and the DMN mask) as a whole. These network masks were then broken into individual region masks of areas forming the networks. Regions of the auditory and sensorimotor systems were also assessed for comparative purposes. ROI analysis was conducted on thresholded (*t* = 2.8), voxel-wise uncorrected *t*-maps. BOLD activation analysis is divided into analyses of activation strength and activation extent. Strength of activation refers to the average intensity surviving a specific statistical threshold (*t* = 2.8 corresponding to a *p*-value of 0.005) within a region, while extent of activation refers to the count of voxels that comprise the activation strength. The average Student's *t*-value of the voxels within the boundaries of the defined masks served as a measure of activation strength, and the number of activated voxels was used as the measure of extent of activation.

### Task-induced functional connectivity analysis

Two 6-mm radius ROIs were created based on coordinates of peak activation in the younger subjects' group BOLD activation map following the phonemic verbal fluency task. Left IFG (Broca's area) seed [−42, −3, 30 (TLRC)] was based on peak positive activation, and right pC/PCC seed [6, −60, 32 (TLRC)] was based on peak negative activation (or maximal deactivation). Whole brain voxel-wise connectivity analyses with those two seed regions were assessed following the same cluster-wise correction for multiple comparisons as determined previously by Monte Carlo simulations. Moreover, the functional connectivity analysis is divided into intensity and extent of connectivity as well, similarly to the method introduced in Section Task-Induced Functional Connectivity Analysis. The intensity of connectivity was calculated as the average *Z*-value within the boundaries of the specific regions surviving certain statistical threshold (*t* = 2.8 corresponding to a *p*-value of 0.005), while extent of connectivity accounted for the voxel count reaching such intensity of connectivity. Task-induced functional connectivity between Broca's area (TLRC [−42, 3, 30]) and regions of the auditory and sensorimotor systems were also assessed for comparative purposes.

### Psychophysiological interactions analysis

Aside from BOLD activation and functional connectivity analyses, psychophysiological interactions analysis (PPI; Friston et al., 1997) was implemented to test the interactions between the language network and the default-mode network in the different psychological context of task and rest blocks among the different populations of young, middle-aged, and old. More specifically, we examined the influence of condition (onset/end of task block) on the effect of the seed region on the target regions, the connectivity between the language network and the DMN. PPI analysis was performed with both left IFG and right pC/PCC masks as seeds.

## Results

### Subjects' characteristics and behavioral performances

Enrolled participants were categorized into three distinct groups: young adult subjects (≤ 30 years), middle-aged subjects (between 50 and 59 years) and older adult subjects (≥60 years), with each age distribution within category distinctively different from the next. Details and statistical significance are presented in Table 1. Other characteristics such as sex, handedness, and education were also assessed, and no significant differences were discerned between the groups.

Out-of-scanner behavior testing consisted of implementation of a phonemic verbal fluency task similar to the one carried inside the scanner, as the primary measure of behavioral performance. Participants were asked to vocalize their answers within their allotted time of 1 min for each of the 3 letters: F-A-S. There was no observation of differences in terms of subjects' performance in the task after normalization of the scores adjusted for age and education, though raw scores illustrated higher scores in the older participants. The lack of behavioral differences between the groups suggests a ceiling effect in the performance of the language production verbal fluency task, which persisted across the different age groups.

### Task-relevant network

From the language production task of phonemic verbal fluency, the regions of the left-IFG, left-MFG, and SMA were consistently activated in all three age groups, consistent with previously reported language-relevant regions (Meinzer et al., 2009). In addition, the right cerebellum was also activated. Activation intensity and extent were extracted from an anatomically-derived mask. Our language network mask included regions of the left inferior frontal gyrus (IFG) −pars triangularis and pars opercularis− (Broca's area), the left middle frontal gyrus (MFG) and inferior frontal gyrus (IFG), the posterior section of the left superior temporal gyrus (STG) (Wernicke's area), and the left supplementary motor area (SMA), as identified and traced using the AFNI program *whereami* based on the CA-N27-ML template. These anatomical masks were mirrored on the contralateral hemisphere to provide a global symmetrical network of the language system. These regions were also verified to be part of the language network by functional connectivity. To assess functional connectivity, we opted for a seed-based approach, where oscillations across the whole brain are assessed against oscillations of a seed-region, in this case voxel of maximal activation during VF task of the left IFG, a core component of the verbal fluency process (Schlosser et al., 1998; Gaillard et al., 2003).

#### Task-induced activation

We refer to task-induced activation as the activation elicited with the performance of the language production task. Activated regions included the left-IFG, left-MFG, and SMA in all three of the population age groups (Figure 1). The right cerebellum was also activated. While differences in the activation pattern between the young and middle-aged subjects were not apparent, the older adults group demonstrated considerable changes (*p* < 0.05), with activations encompassing the contralateral hemisphere. Region-of-interest analysis over bilateral language network mask demonstrated an increase of cortical activation in the participants over the age of 60 (Figure 1, right), predominantly driven by the recruitment of the contralateral hemisphere, more specifically the contralateral homolog, as well as a posterior region over the ipsilateral inferior parietal cortex, possibly Wernicke's area. Intensities of activation within each of the investigated regions of the bilateral language network did not exhibit significant variation between the young and the older groups. But a larger volume of the cortex was being activated in the left hemisphere as well as the right hemisphere in the older adults group, driving the overall increase in cortical activation. Interestingly, a decrease in intensity was observed in participants in their fifties (the middle-aged group) that was lower than the young and the older adult groups.

Activation extent, as recorded by the count of activated voxels surviving a specific statistical threshold (*t* = 2.8), accounted for a step-wise increase in relation to age in the contra-lateral cortical regions (right-IFG, right-MFG, right-SMA). In the cerebellar cortices, a decrease in activation extent in the right hemisphere was present, while in the left cerebellum increases in both intensity and extent were recorded. Weak correlations were found between activation extent over these contralateral regions and age: right-IFG (*R*^2^ = 0.07, *p* = 0.05), right-MFG (*R*^2^ = 0.06, *p* = 0.08), right-pSTG (*R*^2^ = 0.06, *p* = 0.06), right SMA (*R*^2^ = 0.09, *p* = 0.03), and left cerebellum (*R*^2^ = 0.09, *p* = 0.02). Correlations with age for extent of activation of ipsilateral regions were not significant. Stronger correlations were found between activation of the left-IFG to those of the recruited regions: right-IFG (*R*^2^ = 0.36, *p* < 0.001), left-MFG (*R*^2^ = 0.58, *p* < 0.001), left-pSTG (*R*^2^ = 0.36, *p* < 0.001), and right-MFG (*R*^2^ = 0.11, *p* < 0.01). Correlation statistics were not corrected for multiple comparisons.

#### Connectivity within task-relevant system

**Figure 2:**
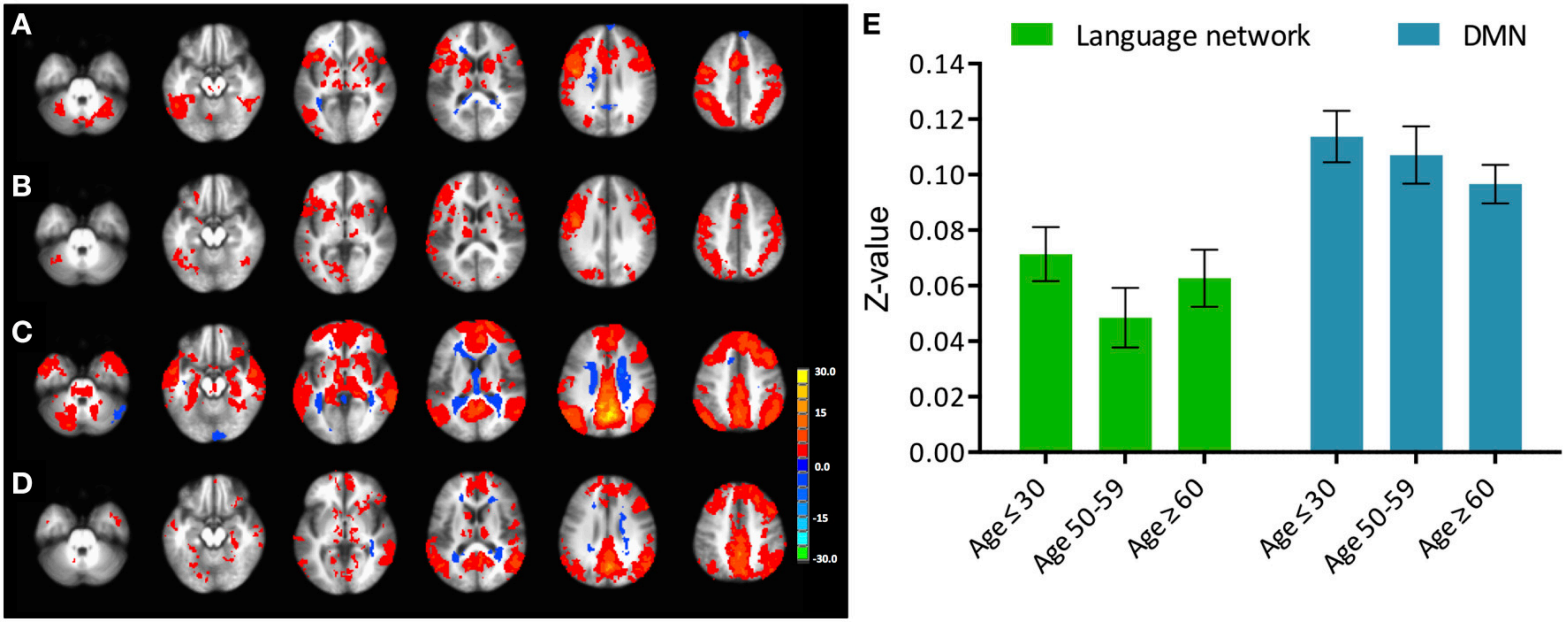
**Phonemic verbal fluency task connectivity maps and quantitative change in functional connectivity between age groups**. (Left) Connectivity maps from **(A)** left IFG seed in young (≤ 30 years), **(B)** left IFG seed in older adults (≥60 years) subjects; **(C)** pC/PCC seed in young (≤ 30 years), **(D)** pC/PCC seed in older adults (≥60 years) subjects, **(E)** mean connectivity score within the language network and the DMN for each group. No differences were observed in connectivity in either system.

The verbal fluency task-positive network was derived by regions expressing synchronicity of their temporal oscillation pattern to that of the seed region placed in the left IFG (or Broca's area). In the young individuals, the extracted functional regions of the network comprised of bilateral IFGs, bilateral MFGs, SMA, bilateral SPLs, bilateral posterior STGs, and bilateral lobes of the cerebellum (Figure 2, left panel). Maps from the two older groups (between 50 and 60, and over 60) did not differ from that observed for the young (≤ 30).

**Figure 3:**
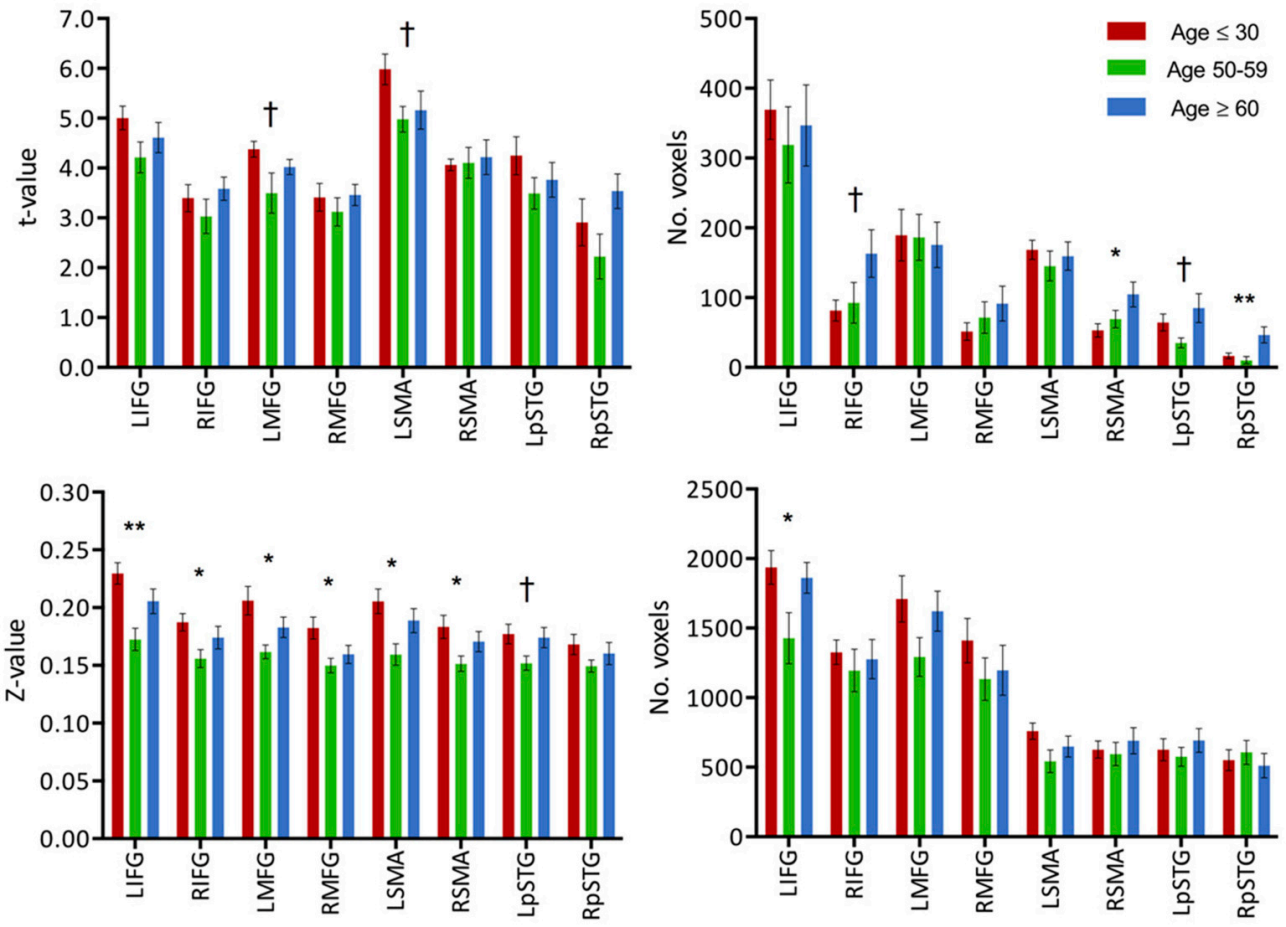
**(Top) Intensity and extent of activation over the task-positive network**. Mean intensity (left) and cluster extent (right) within ROIs of the task-positive (language) network, showing areas actively implicated in the language function and their homologs. **(Bottom)** Functional connectivity strength and extent of connectivity over the task-positive network. Mean functional connectivity strength (left) and extent of connectivity (right) to left IFG for individual regions of the language network and their homologs. Statistical values are generated from ANOVAs between the three population groups for each of the ROIs. (^**^Significant at *p* < 0.005, ^*^Significant at 0.005 < *p* < 0.05; ^†^trending).

Similar to the analyses of task-induced activation, a measure of the strength of the correlation and another of the extent of correlation were assessed. Strength of correlation within this network varied across the populations with the highest correlation strength in the young group, and the lowest correlation strength in the late middle age group. The older adult group (≥60 years of age) (Figure 2E) exhibited lower connectivity strength than the young group, but demonstrated higher connectivity strength than the middle-aged group (Figure 2). This pattern was consistent across the 10 non-overlapping, pre-defined ROIs forming the network (Figure 3, bottom). Differences between groups in extent of connectivity were not significant with the exception of the increase in extent of connectivity over the left IFG itself (Figure 3).

### Task-negative system

We referred to task-induced deactivation as activity of lower intensity than baseline during performance of the task proper. In all three population age-groups, task-induced deactivation was demonstrated in regions of the PCC, bilateral IPL, and the medial PFC (Figure 1), known regions of the default-mode network. Those regions, all previously shown to be greatly involved in the default-mode network (DMN), were used to form a mask of the task-negative system, which we will refer to as the DMN for simplification purposes.

#### Task-induced deactivation

**Figure 4:**
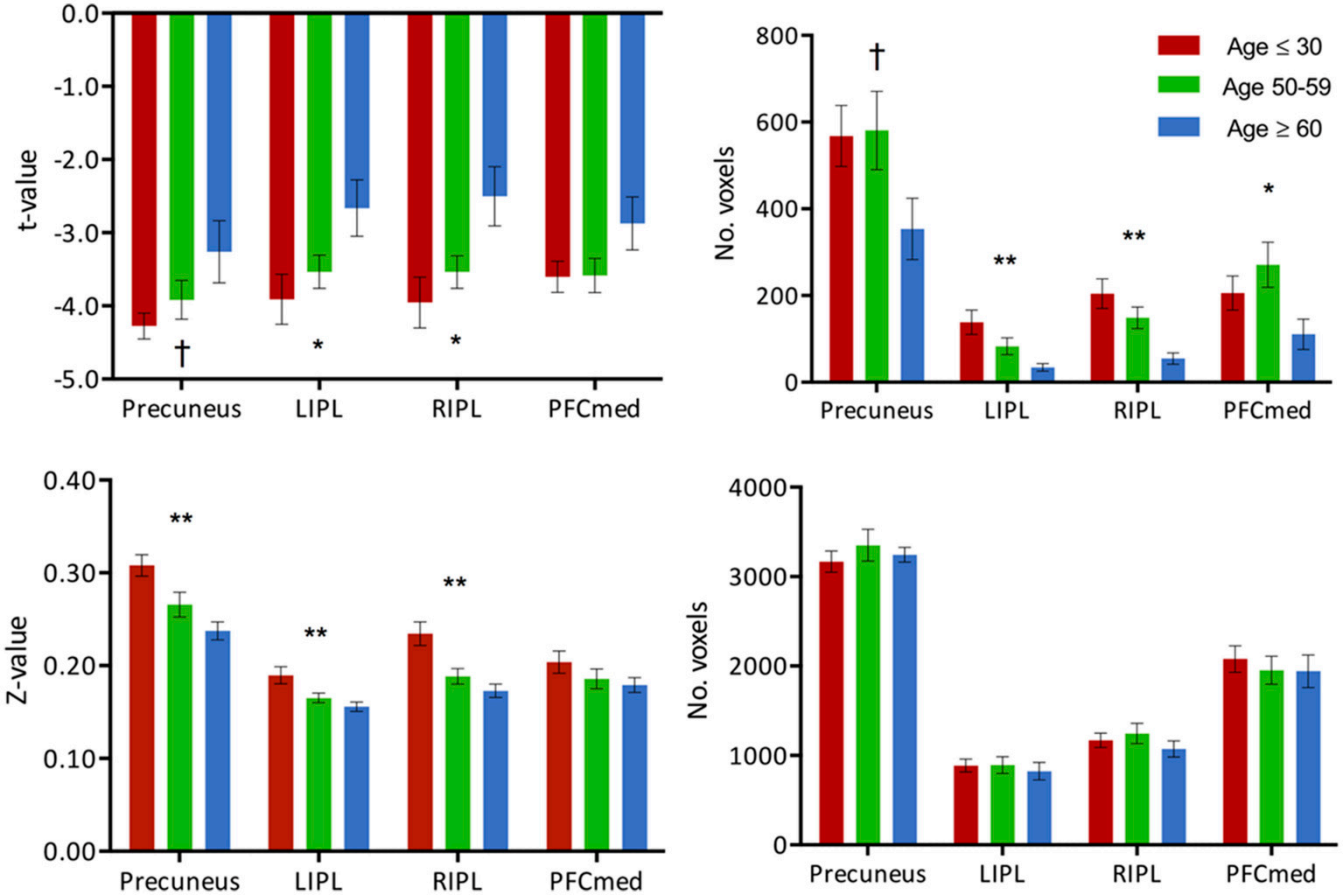
**(Top) Intensity and extent of activation over the task-negative network**. Mean intensity (left) and cluster extent (right) within ROIs of the task-negative network (DMN): pC/PCC, bilateral IPL and medial PFC. **(Bottom)** Functional connectivity strength and extent of connectivity over the task-negative network. Average connectivity strength (left) and extent of connectivity (right) to the pC/PCC seed (TLRC [6, −60, 32]) within regions of the DMN. Connectivity strength (left) for regions of the DMN present an apparent step-wise reduction with age while extent of connectivity (right) is similar across groups for each region. Statistical values are generated from ANOVAs between the three population groups for each of the ROIs. (^**^Significant at *p* < 0.005, ^*^Significant at 0.005 < *p* < 0.05; ^†^trending).

Task-induced deactivation was strongest in the group of young adults, followed by the middle-aged, then older adults. While the difference between the young and middle-aged individuals were not significant, the reduction of such deactivation in the older adults was significant (*p* = 0.015) compared to the group of young adults (Figure 1). Similar to the analysis of task positive language network, ROI analysis of cortical deactivation intensity and extent were carried out. However, in contrast to the language network, regions of the DMN presented signs of age-related changes in both intensity and extent (Figure 4, top). All four tested regions (pC/PCC, medial PFC, left and right IPL) demonstrated reduction in both features, contributing to the overall reduction in DMN cortical deactivation, with the reduction in extent of deactivation in the pC/PCC region being the most significant.

The two measures (intensity and extent of activation) over the DMN ROIs were also tested for correlation with age, but only showed weak relationships: (a) intensity: left IPL (*R*^2^ = 0.1, *p* = 0.018), and right IPL (*R*^2^ = 0.14, *p* = 0.004); (b) extent: left IPL (*R*^2^ = 0.22, *p* < 0.001), and right IPL (*R*^2^ = 0.24, *p* < 0.001). Age correlations to pC/PCC intensity (*R*^2^ = 0.05, *p* < 0.07) and medial PFC intensity (*R*^2^ = 0.05, *p* = 0.11) proved to be weak to non-significant. Correlation between deactivation over the pC/PCC and activation of the task-relevant left IFG were not significant either (*R*^2^ = 0.04, *p* = 0.15).

#### Functional connectivity of the task-negative system

The task-negative network was derived by regions expressing synchronicity of their temporal oscillation pattern to that of the seed region located in the right pC/PCC, during performance of the verbal fluency task. For the evaluation of the task-negative system, a seed was placed in known core region of the DMN, over the right pC/PCC complex (Raichle et al., 2001; Buckner et al., 2008; Andrews-Hanna et al., 2010), in the region of maximal deactivation during performance of the VF task.

Analysis of functional connectivity with the right pC/PCC seed elicited a functional network comprising of medial PFC, pC/PCC complex, bilateral IPL (forming the DMN), and the regions of bilateral MFG in the young participants (Figure 4, bottom). The overall patterns of network connectivity did not differ across the groups with similar maps of correlated regions. However, the strength of correlation within the system degraded with increasing age groups. The medial PFC exhibited the least difference between the groups. The extent of the connectivity showed no differences, with consistent voxel counts in all four regions of interest, the pC/PCC, left and right IPL, and mPFC.

### Psycho-physiological interactions (PPI)

Context-dependent correlation analysis or generalized PPI is to account for the effect of task/condition/stimulus on the connectivity model, the interaction between the psychological effect (task/condition/stimulus) and the neuronal response (physiological effect). Here, we examined the influence of condition (onset/end of task block) on the effect of the seed region on the target regions, the connectivity between the language network and the DMN. We conducted two separate PPI analyses, one with left IFG as the seed region, another with the pC/PCC as the seed region.

#### Context-dependent correlation with left IFG as seed

**Figure 5:**
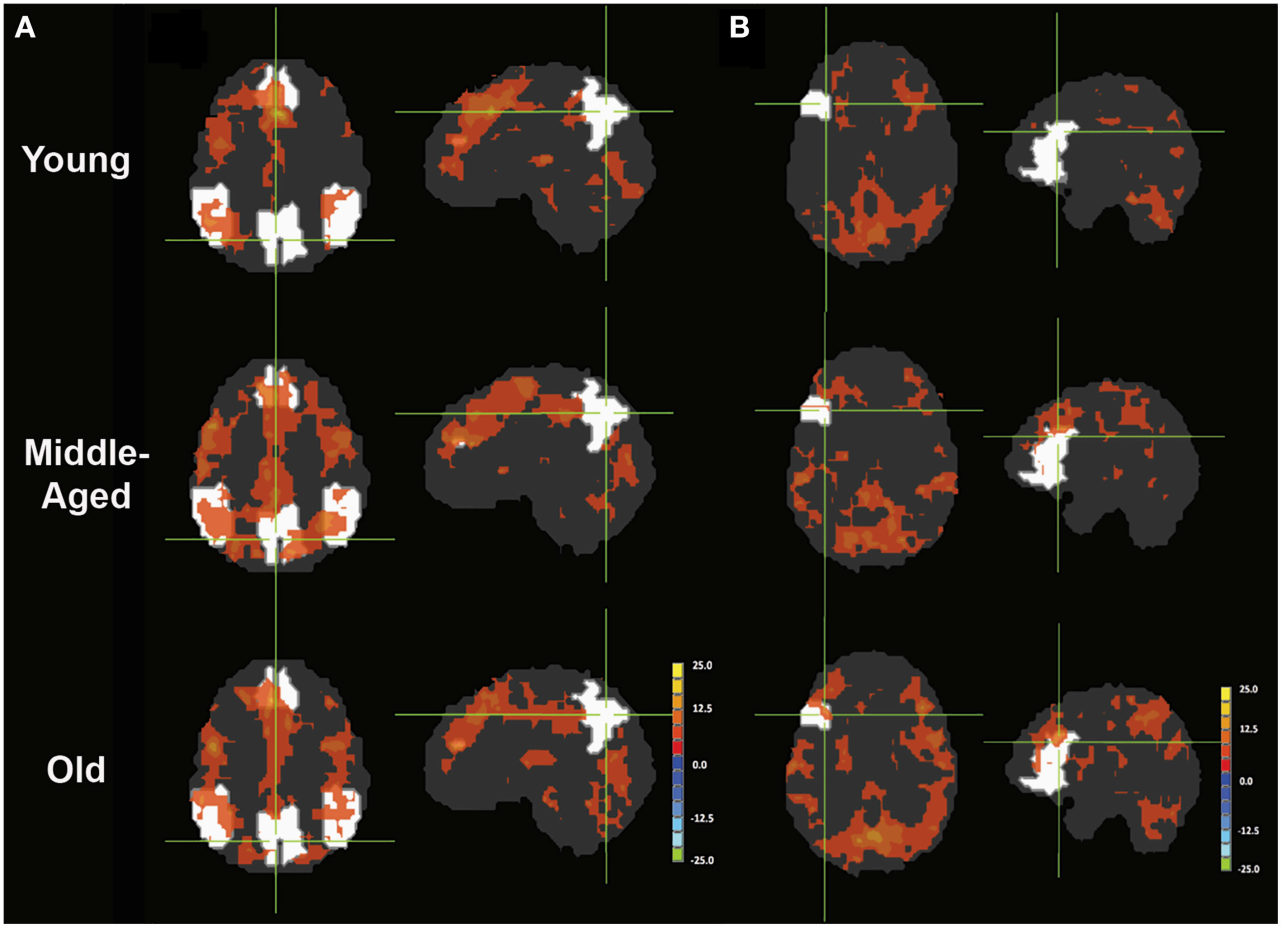
**PPI maps representing the context-dependent interaction between two systems**. A- PPI with seed placed in left IFG with DMN mask underlay (white). B- PPI with seed placed in right pC/PCC with left IFG mask underlay (white). Maps are presented with threshold *p* = 0.00001, with 165 contiguous voxels.

In the young healthy adults, psycho-physiological interaction analysis of the task condition (on/off of the task block) demonstrated a positive increase in connectivity between the seed region of the left IFG and many regions (Figure 5A). Positive condition-to-connectivity interactions were observed in the SMA, the left IFG, left/right parietal lobules, thalamus, and ventral occipital areas, consisting of regions becoming more synchronized with the onset of the task block. Each PPI maps are presented at individual *p* < 0.00001, α = 0.05, with minimum cluster size of 165 contiguous voxels.

**Figure 6:**
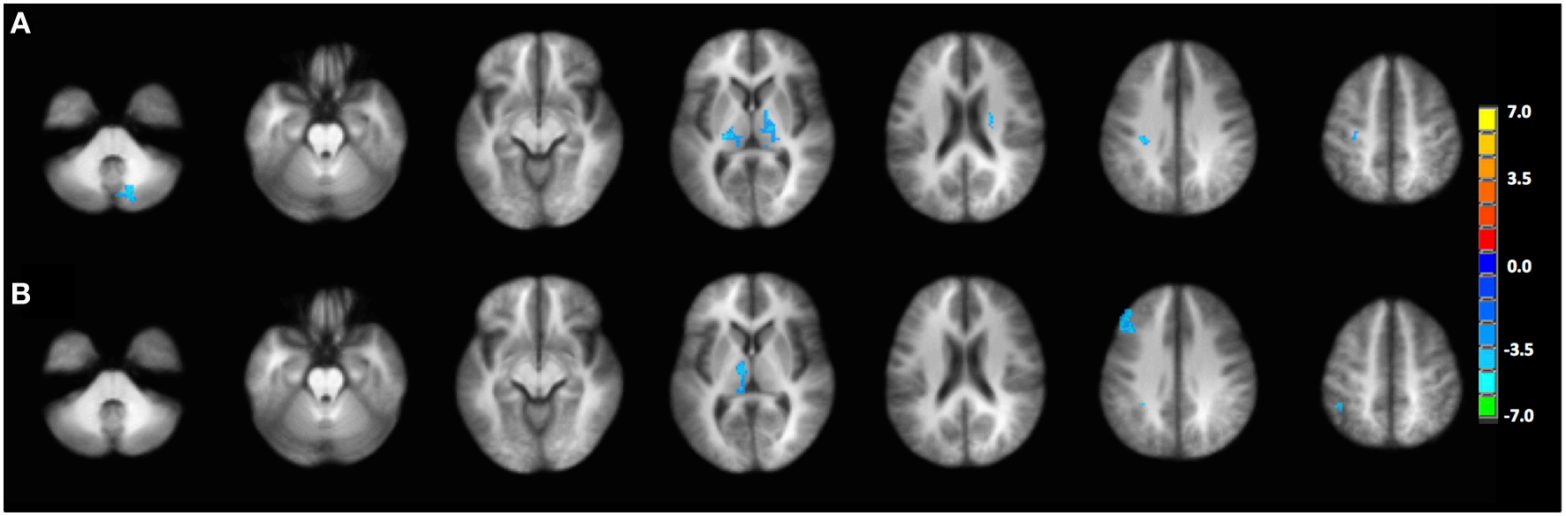
**Statistical difference between the young group and the old group in terms of (A) PPI interaction with left IFG as seed region, (B) PPI interaction with right pC/PCC as seed region**. Statistical maps are shown at *p* = 0.005.

The PPI maps of the middle-aged and of older groups exhibited more extensive correlation, with the inclusion of right MFG, and the cingulum. However, most of those differences between populations were not statistically significant. Only regions of the left and right thalamus, right cerebellum, and left post-central demonstrated significance between the young and the old groups with blue representing elevated context-dependent interaction (Figure 6A and Table 2). Of note is the lack of interaction between the task-relevant system with the PCC, the core region of the DMN.

**Table 2:** **Clusters of significance for the contrast of young vs. older subjects. Obtained at *p* = 0.005, minimum of 65 voxels/cluster**.

**PPI with left IFG as seed**					
**Cluster**	**Peak coordinates (TLRC-RAI)**			**Size**	**Regions**
	***X***	***Y***	***Z***	**(voxel count)**	
1	−18	15	0	228	R thalamus
2	21	21	6	98	L thalamus
3	−15	66	−36	85	R cerebellum
4	27	27	33	68	L postcentral
**PPI with right pC/PCC as seed**					
**Cluster**	**Peak coordinates (TLRC-RAI)**			**Size**	**Regions**
	***X***	***Y***	***Z***		
1	9	12	6	95	L thalamus
2	39	−21	33	76	L MFG
3	6	−21	39	68	L/R cingulate
4	45	45	45	66	L IPL

#### Context-dependent correlation with right pC/PCC as seed

Alternatively, PPI analysis with the pC/PCC region as a seed, demonstrated influence of the task condition (on/off of the task block) on the connectivity of the pC/PCC to the left/right MFG, the PCC, the precunueus, and the right IPL (Figure 5B). Figure 5B also showed very limited context-dependent interaction with the left IFG seed and region of the left IFG in general (white mask). Difference between population groups did not vary extensively, with limited expansion of the pre-existing regions found in the map of the young healthy adults. Statistical differences were found in the left thalamus and left MFG (Figure 6B and Table 2).

### Two additional systems “irrelevant” to VF task

**Figure 7:**
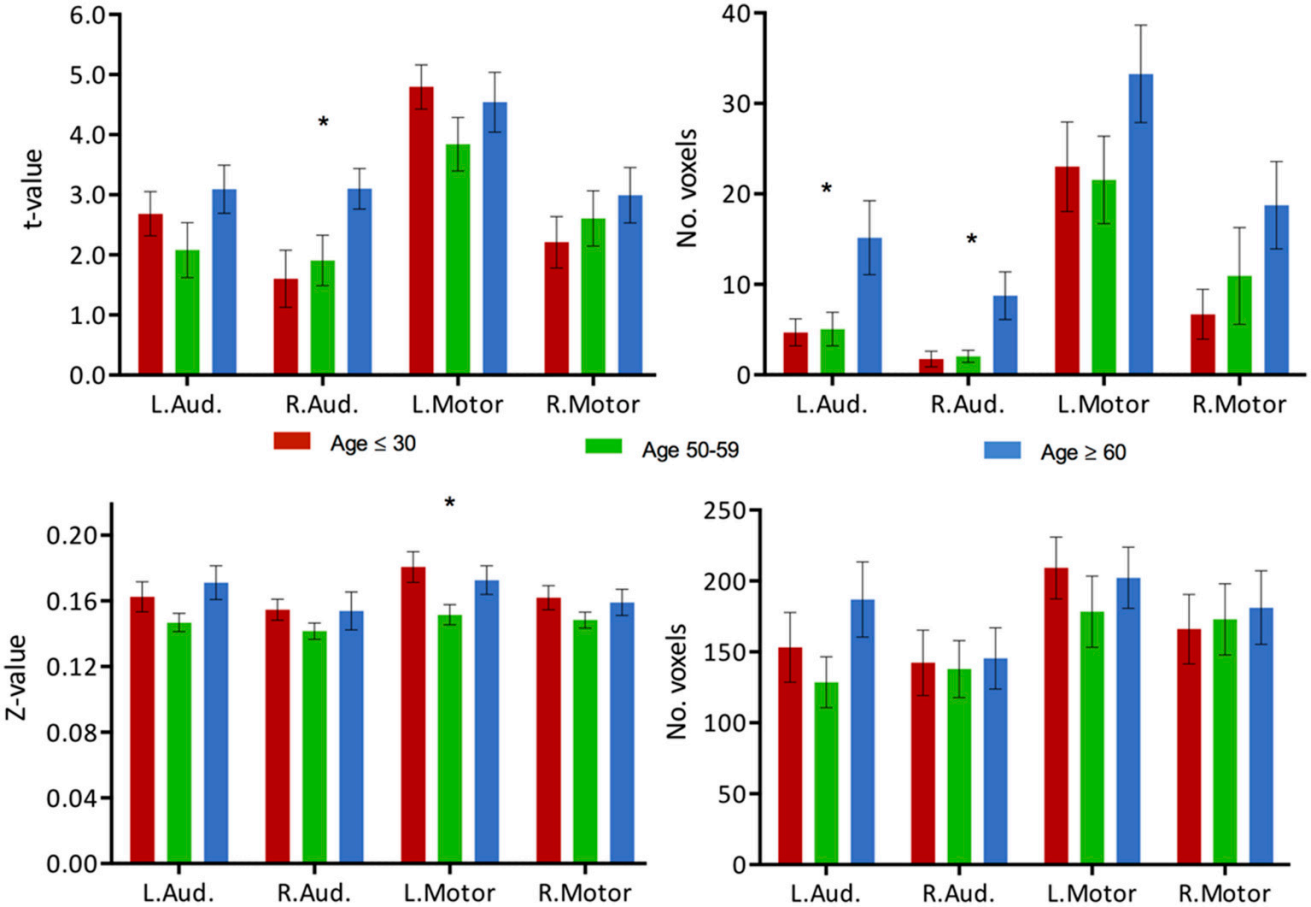
**Task activation and functional connectivity over the motor and auditory networks. (Top)** Mean intensity (left) and cluster extent (right) within ROIs of the auditory and motor networks in a phonemic VF task in young (red), middle-aged (green) and older adults (blue). **(Bottom)** Mean functional connectivity strength (left) and extent of connectivity (right) to Broca's area (TLRC [−42, 3, 30]) for individual regions of the auditory and motor networks. Statistical values are generated using a one-way ANOVA test, uncorrected for multiple comparisons. (^*^Significant at *p* < 0.05).

For comparative purpose, we examined the auditory and motor systems, two task-“irrelevant” networks to language production in order to test the hypothesis of a failure of inhibition of the cortical system. Both systems demonstrated increase in activation with age, in particular in the older age group, despite not being systems actively recruited during performance of the VF task. The increase in activation intensity reached significance in the right auditory cortex, while an increase in the extent of activation was observed in both left and right auditory cortex (Figure 7, top, ANOVA, *p* < 0.05). Elevated levels of activation (intensity and extend) were also found in the motor regions, but did not reach significance. In regards to connectivity, very limited connectivity differences were found in those task-“irrelevant” systems. Only a difference in the intensity of the connectivity between the left motor region with Broca's area (TLRC [−42, 3, 30]) was recorded (ANOVA, *p* < 0.05), with the difference describing a reduction of strength of connectivity only in the middle-aged group (Figure 7, bottom).

## Discussion

Although language function is traditionally considered a left lateralized process, there is some evidence for an active role of the right hemisphere homologous regions in certain aspects, such as contextual processing and the appreciation of metaphors (Vigneau et al., 2011; Yang, 2014), or overlearned categories (Birn et al., 2010). These findings suggest a potential involvement of the right hemisphere, more precisely of the right hemisphere homolog in language functions as well. Similar to the memory and motor system, task-based functional MRI (fMRI) studies have shown reduction in the lateralization of the network pertaining to various language tasks in healthy older adults. Homolog activations over the right hemisphere have been observed during performance of a verbal fluency task, particularly in older subjects with impaired performance (Meinzer et al., 2012). This shows that a similar mechanism of reduction of hemispheric asymmetry is possible within the language system.

In this study, we revisited the HAROLD model in behavioral-equivalent groups of older, middle-aged, and young adults on a language fluency task, examining both intensity and extent of activation, in order to provide a more comprehensive understanding of the aging mechanism. We further investigated the level of deactivation on the “task-negative” network of the DMN across the different age groups. Additionally, we investigated the changes of functional connectivity, in both strength and extent of the system correlations. Finally, we concluded with an implementation of an analysis of the context-dependent correlation to examine the nature of relationship between the language network and the DMN in order to ascertain whether these networks are indeed anti-correlated.

### Task-positive network

Overall activation of language network regions intensified with aging with a recruitment of the contralateral hemisphere during a phonemic verbal fluency task (Figure 1), which is consistent with previous studies (Meinzer et al., 2009). We further uncovered that this observed increase of cortical activation in the group of older adults was driven by an expansion of the cortical regions recruited rather than an increase in the intensity of activation. This expansion consisted of regions both in the contralateral hemisphere, such as the right IFG, right MFG, right SMA, right STG and left cerebellum, and an expansion in the ipsilateral network with the inclusion of the left STG.

The inclusion of the left posterior STG in the oldest group suggests that this expansion of the recruited regions is not limited to prefrontal homologs, but expands to posterior regions that may not have necessarily been involved during performance of the task in young healthy individuals. These regions however, are within the same functional network as shown in the functional connectivity analysis, predisposing those regions for recruitment if necessary. Additionally, no changes in the mapping of the task-positive network were found (Figure 2), indicating that no novel regions were recruited that did not already pertain to the network previously.

The lack of difference in the VF normalized scores demonstrated a behavioral equivalence among the subjects tested in regards to the language production task of verbal fluency, despite their age difference. While presenting behavioral equivalence in task performance to the young participants, the group of older adults recruited a more extensive network, also associated with an expansion of the activation over the contralateral hemisphere. It was previously observed that with aging, there is a reduction in connectivity within a specialized network such that the network organization was less efficient with decreased connectivity within the key regions of the activated network, compensated by an engagement of more distributed network (Meunier et al., 2009, 2014). The recruitment of the left posterior STG and the regions of the contralateral hemisphere form this more distributed network. Overall behavioral equivalence among the groups may be due to a reorganization of the functional framework underlying those cognitive processes.

Our results support theories of compensation-related utilization of neural circuits hypothesis (CRUNCH; Reuter-Lorenz and Cappell, 2008) and “scaffolding theory of aging and cognition” (STAC; Park and Reuter-Lorenz, 2009), which describes the recruitment of additional structures, proximal or distal, to preserve performance when the neural structures are declining in normal aging, ultimately forging alternative neural circuits. Additionally, our results posit that the compensatory recruitment of areas (homolog and posterior STG) occurs with an expansion of the extent of recruited brain voxels, and not through an increase in peak intensity. This would suggest that age-related compensation occurs through the recruitment of less task-relevant functionally-specific regions, and not from a recruitment of the contralateral homolog alone. The peak intensity of the regions remains more or less unchanged between the young and the older groups.

A pattern consisting of a reduction in degree of BOLD activation (intensity) in the middle-aged followed by a later increase in the older adults (over 60 years of age) in many of the tested task-ROI was also observed. With all groups behaviorally performing at equivalent levels, these results may be due to a cognitive efficiency increase, with a specialization of the area as the extent of the activation presented a tendency to be reduced as well. However, in combination with the functional connectivity findings in the middle-aged, where decreased functional connectivity was found (intensity and extent), the possibility that reduced BOLD activation over those areas as part of an increase in cognitive efficiency is low. Alternatively, the reduced BOLD activation over those areas may reflect a compensatory activation in response to an initial cognitive decline. The activation and connectivity in the participants over the age of 60 years old in our study may reflect a sign of a compensatory mechanism to maintain performance.

### The default-mode network

The task-negative system of the DMN is a network often investigated in task fMRI in aging studies. In young healthy individuals, regions of this network are known to become deactivated during task performance. With aging, this deactivation is reduced, and such finding has been shown to be associated with decline of cognitive performance (Grady et al., 2006; Persson et al., 2007). This led to our parallel investigation of aging on this network, and assessment of whether the previously proposed “anti-correlation” of task-positive and task-negative systems (Persson et al., 2007; Meinzer et al., 2012) persists.

The degree of activation over regions of the DMN uniformly exhibited reduction in intensity and extent of activation across the age groups. This reduction of DMN sensitivity in the older adults is consistent with previously reported decreased activation in resting state (Damoiseaux et al., 2008), and reduced deactivation during task (Grady et al., 2006; Persson et al., 2007). However, in contrast to changes in the task-positive network, changes over the DMN in cortical activation are driven by both intensity and extent of activation with all four tested DMN ROIs exhibiting this reduction of deactivation (Figure 4, top). Strength of the connectivity within the system also decreased, though the extent of the network did not. This finding marked a linear and progressive decline of the network, marking a more general disruption of the system, which is dissimilar to the pattern observed in the task-relevant network, where an initial decline was observed in the middle-aged group with reinforcement of intensity and extent of the ipsilateral ROI accompanied by a gradual increase of contralateral ROI as part of a compensatory mechanism in the older adults. Therefore, we further investigated whether these two systems are “anti-correlation” or the alternate hypothesis of two concurrent independent networks (Buckner et al., 2008; Meinzer et al., 2012).

### Psycho-physiological interactions

Psycho-physiological interactions or context-dependent correlation analysis assesses the effect of task/condition/stimulus on the connectivity model, in other words the interaction between the psychological effect (task/condition/stimulus) and the neuronal response (physiological effect). Here, we examined the influence of condition (onset/end of task block) on the effect of the seed region on the target regions, the connectivity between the language network and the DMN to provide a better understanding of the relationship between the two systems.

The difference in the PPI analysis of the different age groups presented few significant regions with decrease interaction with the effect of condition, residing primarily in the region of the thalamus, the relay center for cortico-cortico synchronization (Singer, 1993; Sherman, 2005; Saalmann et al., 2012). Though not reaching significance in the between-group comparison, the young healthy adults demonstrated a more lateralized effect of task on the interaction between the left IFG and regions within the left cortical hemisphere of language, while middle-aged and older healthy adults presented a more bilateral interaction pattern. This finding mirrors task-induced activation maps (Figure 1), where activations were more restricted to the left IFG, the SMA, and right cerebellum, suggesting high cortical specialization. With advanced age, task-induced activations become observable in the contralateral homolog of the right IFG as well, replicated here in the more bilateral interaction map (Figure 5A). One difference between task-induced activation and the PPI interaction maps is at what time recruitment of the contralateral hemisphere occurs. In the middle-aged group, the subject within the group still exhibited activation lateralized to the left, yet presented a bilateral interaction map, suggesting an early level of recruitment of the contralateral hemisphere.

Regarding the interaction between the two systems (language and DMN), minimal interactions are observed, with the onset of task blocks neither influencing positively or negatively the correlation between left IFG seed and pc/PCC (Figure 5A). Reciprocally, the onset of task blocks neither positively nor negatively influences the correlation between the pC/PCC seed with the left IFG, the main region implicated in the VF task (Figure 5B). Adding to the difference in the pattern of aging on task-induced activation (intensity and extent), with task-relevant network presenting initial decline followed by compensatory reorganization and gradual decline of the DMN, this analysis of psycho-physiological interaction provides evidence of a lack of inter-dependence of the language and DMN systems, or an inter-independence of the two systems. The language system and DMN may potentially be only linked by the need of a reallocation of cortical and functional resources.

### Two additional “task-irrelevant” systems

If indeed the language and DMN are two independent systems, linked by the re-allocation of resources within the cortical system, then the failure to inhibit the DMN in the older healthy individuals would parallel the suppression of other non-relevant systems as well, resulting in an elevated level of activation of these non-relevant systems, ultimately resulting in increased noise in the cortical system (Winterer et al., 2004; Backman et al., 2006). We assessed two other non-relevant systems: an auditory and a motor system. No expansion of the functional connectivity maps to those areas was observed; hence it is unlikely that the network involved in verbal fluency expanded to those regions. Regions of the auditory network presented comparable effect of an increase in BOLD activation vs. a reduction of inhibition in the DMN (Figure 7). Significance of the change in the motor is more limited, potentially due to a partial “irrelevance” of the system to the VF task. The motor system has been implicated in the production of language with elicitation of motor and muscle activation. However, the increase in intensity and extent over the auditory system may present further evidence of a cortex-wide, general failure to disengage and suppress non-relevant activity. The hypothesis of a reduction of inhibitory ability of the cortical system in the aging brain is not discredited. Similarly, in working memory paradigms, the hypothesis of failures of inhibition of non-relevant processes contributing to the cognitive changes observed with age has been previously been put forward (Hasher and Zacks, 1988), and have been suggested to be potentially mediated by a lack of top-down modulatory control exerted by the PFC (Gazzaley et al., 2005; Gazzaley and D'Esposito, 2007). The DMN may very well be comparable to one of such non-relevant processes.

Despite the DMN offering signs of anti-correlation, including a deactivation during task performance and activation during resting-state, but also a reduction of the deactivation accompanying the reduction of task-relevant activation, we did not find plausible correlation suggesting direct relationship between reduced activation over the left-IFG and decreased deactivation of the pC/PCC. It remains possible that perturbation in the DMN is an independent process from the perturbation observed in the task-positive network, which might be reflecting a more general deficit of the brain to properly disengage the networks non-relevant to task. The signs of anti-correlation would therefore be a reflection of the re-allocation of resource among independent systems.

### Study limitations

It is important to acknowledge several limitations in this study. First, two slightly differing scanning protocols were used in the study due to the two MR scanners used, potentially introducing confound to our results. However, such scanner and scanning protocol differences were accounted for as covariate during the analysis. Second, the study was implemented with a non-vocal version of the COWAT task, where participants were instructed to think, but not to vocalize words that start with the letter shown in the center of their visual field, producing no in-scanner behavioral data. The use of a non-vocal version of the task was chosen to minimize head motion, but in retrospect, the availability of in-scanner behavioral data would benefit the interpretation and significance of the observed results. Another limitation resides in the definition of the seed regions in the PPI analysis. Fluctuations within the left IFG and right PCC masks were defined as the seed fluctuations for the PPI analysis. The lack of specificity of those oscillations may have diluted the overall strength of between-regions correlations, but this definition helped in the comparison between the different age groups.

## Conclusion

In a highly lateralized network, such as in language, studies in the literature have demonstrated that the organization of brain activation changes with age, commonly with a recruitment of the contralateral homologous regions along with differences in behavioral performance. Our results suggest reduction of hemispheric asymmetry with aging occurs even when matched in terms of behavioral performance. This occurs through an expansion of the recruited areas rather than an increase in the intensity of the activation. Such expansion involves previously silent regions within the same network, regions that are physically and functionally connected, predisposed to participation. Given that no significant differences were found between groups in verbal fluency task scores, the illustrated reorganization of the functional activity and connectivity pattern may have allowed for the maintenance of task performance despite the increasing age. Furthermore, in addition to the language network, the task-negative network of the DMN experienced changes with the progression of age as well. This network presented lower values not only in terms of both intensity and extent of activation, but also in terms of strength of the correlation within the network. However, the DMN showed more gradual change with age rather than an abrupt change, which can be interpreted as an increased network modulation with aging. This pattern of activity differs from that observed in the task-positive network where a transient reduction in the middle-aged was found. In addition, we did not find plausible correlation suggesting direct relationship between reduced activation over the left-IFG and decreased deactivation of the pC/PCC from our context-dependent analysis, suggesting that changes over these two networks may not be directly associated.

## Author contributions

CL and CG conceived and designed the experiments. CL and CG analyzed the data and wrote the manuscript. CG processed the data. VN, TM, DF, RB, MM, and VP contributed analysis tools and provided study directions.

### Conflict of interest statement

The authors declare that the research was conducted in the absence of any commercial or financial relationships that could be construed as a potential conflict of interest.
